# Type 2 Diabetes in Non-Alcoholic Fatty Liver Disease and Hepatitis C Virus Infection—Liver: The *“Musketeer”* in the Spotlight

**DOI:** 10.3390/ijms17030355

**Published:** 2016-03-09

**Authors:** Stefano Ballestri, Fabio Nascimbeni, Dante Romagnoli, Enrica Baldelli, Giovanni Targher, Amedeo Lonardo

**Affiliations:** 1 Operating Unit Internal Medicine, Pavullo General Hospital, Azienda USL Modena, ViaSuore di San Giuseppe Benedetto Cottolengo, 5, Pavullo, 41026 Modena, Italy; stefanoballestri@tiscali.it; 2 Outpatient Liver Clinic and Operating Unit Internal Medicine, NOCSAE, Azienda USL Modena, Via P. Giardini, 1355, 41126 Modena, Italy; fabio.nascimbeni@libero.it (F.N.); danter1@alice.it (D.R.); 3 Department of Biomedical, Metabolic and Neural Sciences, University of Modena and Reggio Emilia, Via P. Giardini, 1355, 41126 Modena, Italy; enrica.baldelli@unimore.it; 4 Section of Endocrinology, Diabetes and Metabolism, Department of Medicine, University and Azienda Ospedaliera Universitaria Integrata of Verona, Piazzale Stefani, 1, 37126 Verona, Italy; giovanni.targher@univr.it

**Keywords:** epidemiology, cirrhosis, clinical implications, direct acting antivirals, fibrosis, insulin resistance, hepatocellular carcinoma, NASH, pathophysiology

## Abstract

The pathogenesis of type 2 diabetes (T2D) involves chronic hyperinsulinemia due to systemic and hepatic insulin resistance (IR), which if uncorrected, will lead to progressive pancreatic beta cell failure in predisposed individuals. Non-alcoholic fatty liver disease (NAFLD) encompasses a spectrum of fatty (simple steatosis and steatohepatitis) and non-fatty liver changes (NASH-cirrhosis with or without hepatocellular carcinoma (HCC)) that are commonly observed among individuals with multiple metabolic derangements, notably including visceral obesity, IR and T2D. Hepatitis C virus (HCV) infection is also often associated with both hepatic steatosis and features of a specific HCV-associated dysmetabolic syndrome. In recent years, the key role of the steatotic liver in the development of IR and T2D has been increasingly recognized. Thus, in this comprehensive review we summarize the rapidly expanding body of evidence that links T2D with NAFLD and HCV infection. For each of these two liver diseases with systemic manifestations, we discuss the epidemiological burden, the pathophysiologic mechanisms and the clinical implications. To date, substantial evidence suggests that NAFLD and HCV play a key role in T2D development and that the interaction of T2D with liver disease may result in a “vicious circle”, eventually leading to an increased risk of all-cause mortality and liver-related and cardiovascular complications. Preliminary evidence also suggests that improvement of NAFLD is associated with a decreased incidence of T2D. Similarly, the prevention of T2D following HCV eradication in the era of direct-acting antiviral agents is a biologically plausible result. However, additional studies are required for further clarification of mechanisms involved.

## 1. Introduction

### 1.1. Definitions

Type 2 diabetes (T2D) identifies the more prevalent category of diabetes mellitus and is due to a progressive insulin secretory defect in the background of insulin resistance (IR) [1]. T2D is typically found in obese and overweight middle-aged individuals though the age of its initial manifestation has now been observed shifting towards adolescents and even children [2].

Non-alcoholic fatty liver disease (NAFLD) describes a cluster of hepatic disorders predominantly (though not exclusively) characterized by fatty changes with or without ballooning degeneration and fibrosis (*i.e*., simple steatosis, steatohepatitis (NASH) and advanced fibrosis), which may evolve into cirrhosis (NASH-cirrhosis will typically lose fatty changes) and hepatocellular carcinoma (HCC); NAFLD is commonly observed in insulin-resistant, dysmetabolic individuals without excessive alcohol consumption and other competing etiologies of liver disease [3,4]. There is now compelling evidence that NAFLD is a multisystem disease associated with a wide range of extra-hepatic manifestations, notably including, among others, IR, dysglycemia and premature atherosclerosis [5,6].

Hepatitis C virus (HCV) is a small enveloped RNA virus belonging to the genus Flaviviridae, of which six different genotypes are recognized and which is transmitted via the parenteral route [7]. In several countries there have been two major HCV epidemics. The first one (mostly sustained by genotype 1 HCV) took place in the 1960s as a result of HCV being transmitted via medical procedures. The second one (predominantly due to genotype 3 HCV) occurred in the 1980s owing to needle-sharing practices among intravenous illicit drug users [7].

The natural course of HCV infection is variable and modulated by the interaction of host and viral factors. Of concern, the chronicity rate following acute infection approximates 85%, giving way to dreadful *sequelae*, such as chronic hepatitis, cirrhosis, end-stage liver failure and HCC [7]. Similarly to NAFLD, HCV infection is increasingly identified as a systemic disease which may be conducive to metabolic disorders (including IR and T2D) and premature atherosclerosis [8].

### 1.2. Epidemiology and Burden of Type 2 Diabetes

The world prevalence of T2D was estimated to be 6.4% in 2010 and is projected to rise to 7.7% in 2030 [9]. Recent estimates of T2D prevalence in the main five European countries (France, Germany, Italy, Spain and UK) ranged from 4.8% in Italy to 8.9% in Germany, with rates increasing steadily over the past two decades in all these countries. Of concern, in these European countries the total direct medical costs of T2D in 2010 were estimated to range from 5.45 billion euros in Spain to 43.2 billion euros in Germany, with hospitalizations due to T2D-related complications accounting for the greatest proportion of these costs [10]. In the USA, T2D now affects up to 8%–10% of adults in the general population in whom it increases up to four-fold the risk of major cardiovascular events and is the leading cause of blindness, chronic kidney failure and non-traumatic lower extremity amputations [11]. In 2007, T2D posed on society a cost as high as 174 billion dollars in the USA [12]. Of concern, this already alarming prevalence of T2D is predicted to be increasing in all age groups, making it urgent for clinicians, researchers and health authorities to gain a better understanding of the pathophysiology of T2D aimed at preventing the further spread of its disastrous pandemic [13].

### 1.3. Liver and Type 2 Diabetes: Historical Overview

In the past, clinicians and pathologists viewed the hepatic fatty changes as a histological correlate of the coexistence of T2D and obesity (the so-called “*diabesity*”) [14], a conclusion which has been fully supported by contemporary studies [15]. Stated otherwise, the liver was essentially regarded as a target organ affected by either concurrent or pre-existent “*diabesity*”.

More recently, however, this perspective has been fully overturned. Several studies have now exhaustively proven that hepatic steatosis precedes the development of T2D and Metabolic Syndrome (MetS) in a large proportion of cases [16,17,18]. *In tandem*, epidemiological evidence has also suggested that HCV infection almost doubles the risk of incident T2D compared to both HBV infection and virus-free individuals [19]. This is of outstanding interest given that HCV infection is a systemic disease [20] that often exhibits hepatic histological changes of variable severity, including hepatic steatosis, which makes it conceptually similar to NAFLD [7,21]. Excitingly, a cure for HCV has recently become available with direct acting antivirals [22,23,24].

Collectively, all the above findings support the notion that there is a causal, bi-directional link between NAFLD and T2D [25]; that HCV infection is a diabetogenic condition [19]; and that T2D is potentially preventable by curing NAFLD [26] and HCV infection [27].

### 1.4. Aim of the Review and Evidence Acquisition

**Figure 1:**
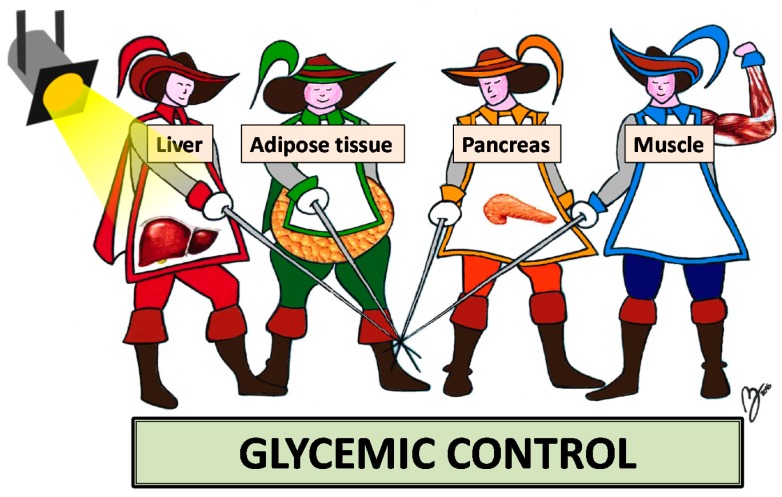
The “*four musketeers*” fighting for maintaining glucose homeostasis. Under normal conditions, muscle and pancreas improve glycemic control. However, an expanded adipose tissue will usually lead to dysglycemia. Similarly, fatty changes occurring in the liver will result in the development of insulin resistance. Hence, this review article puts the liver in the spotlight.

The liver, the skeletal muscle and the pancreas are the anatomic basis of IR and they have collectively been alluded as the “*three musketeers*” [28]. Along with these three organs, the adipose tissue is the “*fourth musketeer*” which is implicated in the pathogenesis of IR (Figure 1) [29]. Over the last decade, the liver has been put in the spotlight of research and our group has been gaining particular interest in the association between the steatotic liver and risk of incident T2D. Accordingly, the main purpose of this article was to review data linking T2D with either NAFLD or HCV infection. For each of these two liver diseases, we will discuss systematically the epidemiological burden, the pathophysiologic mechanisms and the clinical implications.

In order to retrieve pertinent articles, the PubMed database was extensively searched for reports published through 31 January 2016. To this end, we used the following keywords “nonalcoholic fatty liver disease” or “NAFLD” combined with “insulin resistance”, “type 2 diabetes” or “diabetes”. The same keywords were used to identify those articles in which “insulin resistance”, “type 2 diabetes” or “diabetes” were combined with either “HCV” or “hepatitis C virus”.

The selection of articles was performed based on agreement among the authors. Cross-references were taken in consideration based on the authors’ judgment.

## 2. NAFLD and Type 2 Diabetes

### 2.1. Epidemiology

**Figure 2:**
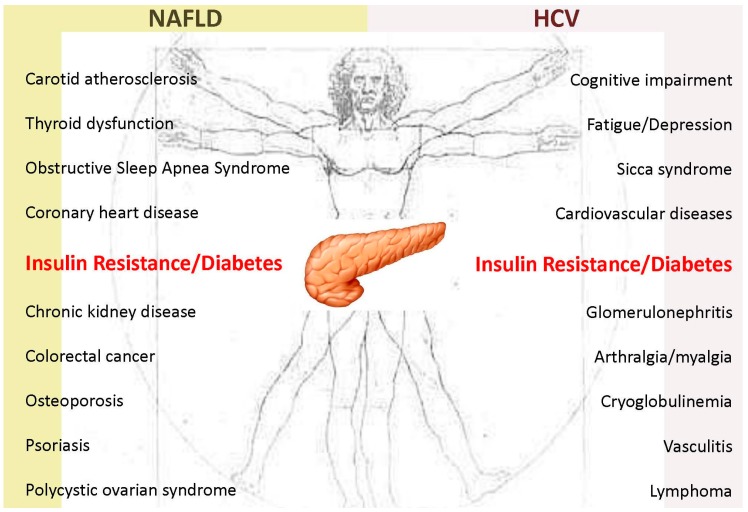
The spectrum of extra-hepatic manifestations and correlates of both non-alcoholic fatty liver disease (NAFLD) and hepatitis C virus (HCV) infection: type 2 diabetes is a shared feature. This figure illustrates the concept that NAFLD and HCV infection are two systemic diseases whose spectrum of clinical manifestations tends to overlap significantly. Type 2 diabetes is a feature shared among the various pathologic conditions included in the NAFLD clinical spectrum [5,6,30,31,32,33,34,35,36] as well as in the clinical spectrum of chronic HCV infection [8,37,38].

The wide spectrum of the extra-hepatic manifestations and correlates of NAFLD includes cardiovascular diseases (CVD), chronic kidney disease, colorectal cancer, obstructive sleep apnea syndrome, psoriasis, endocrine disorders, notably including IR/T2D, thyroid dysfunction, polycystic ovarian syndrome and osteoporosis (Figure 2) [5,6,30,31,32,33,34,35,36]. Epidemiological data fully support a bi-directional relationship between NAFLD and T2D [25]. Stated otherwise, NAFLD is associated with established T2D in cross-sectional studies and precedes the development of T2D in follow-up studies [3,16,18].

#### 2.1.1. NAFLD as a “Manifestation” of Type 2 Diabetes

A consistent body of epidemiological evidence supports the conclusion that NAFLD is strongly associated with T2D and that T2D is a major modifier of the epidemiological features of NAFLD [3,39]. For example, the prevalence of NAFLD (assessed by ultrasonography) is approximately 25%–30% in the general adult population, and men outnumber women by 20% to 40%. In patients with T2D, the prevalence of NAFLD is considerably higher (occurring in up to 75% of these patients), and, remarkably, T2D abrogates sex differences among patients with NAFLD [3,39]. The prevalence of NAFLD in patients with T2D ranges widely from 45% to 75% in large hospital-based studies and from 30% to 70% in population-based studies; this wide inter-study variability is largely due to differences in the ethnicity, population characteristics and criteria adopted for the diagnosis of diabetes [39]. The prevalence of histologically diagnosed NASH, *i.e.*, the more rapidly progressive form of NAFLD [40], is estimated to occur in 2%–3% of the general adult population [6]; conversely, it ranges from 56% to 76% in hospital-based studies [41,42] and from 22% to 83% in outpatient cohort-based studies among individuals with T2D [15,43,44]. Notably, a recent study reported a high prevalence of NAFLD (76%) and NASH (56%) in obese T2D patients with normal serum aminotransferase levels [42]. The finding that many T2D patients with NAFLD have fairly normal serum transaminase concentrations is not reassuring given that NASH, advanced fibrosis and even cirrhosis may occur in such patients with “normal” serum aminotransferases [39,45,46]. Taken together, these studies suggest that the “normal” range of serum liver enzymes needs to be lowered to capture more NAFLD cases.

#### 2.1.2. NAFLD as a Precursor of Type 2 Diabetes

Accumulating data from observational prospective studies indicate that NAFLD (as diagnosed by serum liver enzymes or imaging) is strongly associated with an increased incidence of both T2D and MetS [3,45]. Two large meta-analytic studies have provided further evidence for a strong association between NAFLD and increased risk of incident T2D [17,18]. The first of such meta-analyses, published by Musso *et al.*, [17] found an approximately two-fold increased risk of incident T2D among patients with NAFLD. The second one, recently published by our group, confirmed that NAFLD was associated with an almost two-fold increased risk of developing both T2D and MetS over a median period of five years. Worryingly, our meta-analysis is first in suggesting that the risk of developing MetS was much higher in those in whom NAFLD was identified by ultrasonography compared to those in whom NAFLD was identified based on abnormal liver enzymes [18]. In agreement with these findings, a retrospective cohort study by Sung *et al.* [47] showed that individuals in whom ultrasonography-assessed NAFLD developed or worsened over five years had a marked increase in T2D risk, suggesting that more severe NAFLD is associated with a higher risk of incident T2D [47]. Conversely, individuals in whom NAFLD resolved over five years did not show an increased T2D risk [47]. Similarly, a recent retrospective study reported a strong and independent association between NAFLD improvement and reduced incidence of T2D [48]. Moreover, another recent study has shown that non-overweight individuals with NAFLD had a substantially increased risk of incident T2D compared with both overweight and non-overweight NAFLD-free individuals [49]. Finally, the Multi-Ethnic Study of Atherosclerosis [50] has shown that NAFLD, assessed by computed tomography, was associated with an increased risk of incident T2D independent of common risk factors of T2D.

To date, there is a paucity of published data regarding the association between biopsy proven-NAFLD and the risk of incident T2D or MetS. In a retrospective cohort of 129 Swedish adults with histologically confirmed NAFLD and elevated liver enzymes, the baseline prevalence of T2D was 8.5% and approximately 80% of cases developed T2D (58%) or pre-diabetes (20%) at the end of a 14-year follow-up period [51].

In conclusion, a large body of epidemiological evidence supports the notion that the prevalence of NAFLD is remarkably increased in patients with T2D and that NAFLD is closely associated with an increased risk of incident T2D and MetS.

### 2.2. Pathophysiology

The pathogenic mechanisms linking NAFLD and T2D encompass a complex cross-talk among different organ systems, notably including the gut and the nervous system further to the previously alluded “*four musketeers*”: the adipose tissue, the skeletal muscle, the liver and the pancreas.

#### 2.2.1. Remodeling of White Adipose Tissue

Excess visceral adiposity is a key factor in connecting NAFLD and T2D. The expansion of white adipose tissue (WAT) is associated with hypoxia and adipocytes necrosis [52,53,54,55]. The former causes the release of hypoxia inducible factor 1α (HIF1α), while adipocytes necrosis induces infiltration and M1-polarization of macrophages, thus producing WAT dysfunction, inflammation and fibrosis [53,55,56,57,58,59,60,61,62]. Such a WAT remodeling causes a dysregulation of multiple endocrine and lipid storage functions [54,62]. Dysfunctional WAT, in its turn, is associated with an imbalanced cytokine release, *i.e.*, over-production of multiple pro-inflammatory adipocytokines, such as tumor necrosis factor (TNF)-α and monocyte chemoattractant protein-1/C-C chemokine receptor-2 (MCP-1/CCR-2), and reduction of adiponectin, which contribute to worsen local and systemic metabolic derangements [62,63,64,65,66,67,68,69,70,71,72]. Increased interstitial fibrosis in WAT limits adipose tissue expandability [52,53,62]. Reduction in lipid storage capacity also contributes to ectopic lipid accumulation in the liver, skeletal muscles and pancreas where lipotoxicity triggers multiple pathways that hinder insulin signaling [53,62,73,74]. All of these mechanisms may contribute to the development of IR in the adipose tissue with its inherent failure to suppress adipose lipolysis that results in an overflow of free fatty acids (FFAs) to the liver [74].

#### 2.2.2. Role of Skeletal Muscle and Brown Adipose Tissue

Muscle IR, due to intra-myocellular lipid accumulation, occurs early in the course of T2D. It has been suggested that intra-myocellular diacylglycerol (DAG) accumulation activates protein kinase C-θ (PKCθ), which impairs insulin signaling, impeding muscle glucose uptake and leading to increased delivery of glucose to the liver, where it becomes substrate for hepatic *de-novo* lipogenesis (DNL) [74,75,76,77]. Accordingly, it has recently been shown that skeletal muscle steatosis is associated with NAFLD [78].

The myokines, *i.e.*, cytokines produced by the skeletal muscle, have been recently identified as another piece in the interplay linking NAFLD to T2D. Irisin is produced by the skeletal muscle in response to physical exercise and exerts beneficial metabolic effects by recruiting brown adipose tissue (BAT) and triggering thermogenesis [79,80]. Evidence has recently shown that BAT is recruitable post-natally within either WAT or skeletal muscle [81,82,83,84,85]. BAT, through the expression of uncoupling C protein-1 (UCP-1), generates heat and regulates energy expenditure, lipid and glucose metabolism [81,86,87]. For these reasons, both irisin and BAT could be potential targets for the treatment of obesity-related complications. Interestingly, low levels of irisin have been associated with NAFLD and T2D in humans, thus confirming the important role of this myokine in the regulation of energy homeostasis and preservation of a healthy metabolism [88,89,90].

#### 2.2.3. Intrahepatic Fat Accumulation, Hepatic Insulin Resistance and Hepatokines

In NAFLD, steatogenesis results mainly from increased hepatic esterification of FFAs originating from dysfunctional/inflamed WAT (60%), DNL (25%) and diet (15%) [91,92]. Increased lipolysis drives hepatic lipid synthesis through esterification of FFAs and stimulates hepatic gluconeogenesis [92,93,94], thus promoting hepatic IR [74,95]. Muscle IR increases glucose delivery to the liver, thus enhancing DNL. Moreover, dietary monosaccharides, particularly fructose, directly promotes hepatic lipogenesis by increasing sterol regulatory element binding protein 1c (SREBP1c), carbohydrate-responsive element-binding protein (chREBP), peroxisome proliferator-activated receptor (PPAR)-γ coactivator 1-β, and liver X receptor expression [74,96,97,98,99,100,101].

The resulting intrahepatic ectopic storage of lipids has been specifically associated with hepatic IR [74,102]. However, hepatic triglyceride accumulation *per se* is not always harmful. Experimentally, the inhibition of diacylglycerol acyltransferase 2 (DGAT2), an enzyme devoted to hepatocyte triglyceride biosynthesis, decreases hepatic steatosis, but increases markers of lipid peroxidation/oxidant stress, hepatic lobular necro-inflammation and fibrosis [103]. Several lines of evidence support that intrahepatic diacylglycerol (DAG), via activation of PKCε, and ceramides, by impairing Akt2 action and inducing endoplasmic-reticulum stress and mitochondrial dysfunction, are the two major lipid mediators of hepatic IR [74,102,104,105,106,107,108,109,110,111,112,113,114]. Also intracellular localization of lipids in the liver matters [102]. A common single-nucleotide polymorphism of patatin-like phospholipase domain-containing protein 3 (PNPLA3), a lipid droplet protein with triglyceride lipase activity, has been strongly associated with NAFLD, but not with IR [114,115,116,117,118,119,120]. This dissociation between hepatic steatosis and IR is likely due to the accumulation of metabolically inert polyunsaturated triacylglycerols in lipid droplets caused by the PNPLA3 I148M variant [114,121,122]. Other underlying mechanisms clearly implicated in the development of hepatic IR and in the progression of NAFLD are low-grade chronic inflammation, elevated production of reactive oxygen species, activation of unfolded protein response and endoplasmic-reticulum stress, activation of Jun N-terminal kinase (JNK)-1, increased hepatocyte apoptosis and lipo-autophagy [25,92,102,123,124,125,126,127].

Finally, the liver releases several endocrine mediators, the so-called hepatokines, able to impact glucose metabolism, insulin action and secretion. Fetuin-A, which is abundantly secreted by steatotic hepatocytes, mediates IR by inhibiting the insulin receptor, reducing adiponectin expression, and enhancing WAT inflammation and dysfunction, and is independently associated with T2D development [128,129,130,131,132]. More recently, also fetuin-B has emerged as a potentially major player in T2D pathogenesis. Indeed, in their seminal study, Meex *et al.* [133], have shown that 32 hepatokines are differently secreted in steatotic *versus* non-steatotic hepatocytes. By inducing inflammation and IR in macrophages and skeletal muscles, these changes in the secretory products may contribute to the development of metabolic dysfunction in other cell types. These authors have identified higher levels of fetuin-B in the altered hepatokine secretory profile of steatotic livers in obese patients, and have also experimentally demonstrated that fetuin-B impairs insulin sensitivity in myotubes and hepatocytes and causes glucose intolerance in mice [133]. Fibroblast growth factor (FGF)-21 acts as a potent activator of glucose uptake and inhibitor of WAT lipolysis, recruits BAT and is associated with obesity, NAFLD and T2D [134,135,136,137,138,139,140]. Finally, serpinB1 increases pancreatic β-cell proliferation and its deficiency leads to maladaptive β-cell proliferation in IR [141,142].

#### 2.2.4. Gut-Liver Axis

Compelling evidence links gut microbiota, intestinal barrier integrity and NAFLD. Dysbiosis and impaired gut permeability favor the occurrence of endotoxemia and toll like receptor (TLR) 4-mediated inflammation, thereby contributing to the development of IR and other metabolic complications in obese individuals [143,144,145]. Other interactions between the gut and the liver may occur through the production of multiple gut hormones and the entero-hepatic circulation of bile acids that activate farnesoid X receptor in the liver [26].

Although further research is needed, these findings underline the importance of NAFLD as a precursor for the development of hepatic and systemic IR. However, the presence of long-standing IR *per se* is not sufficient to lead to the development of T2D. Gluco-lipotoxicity and genetic factors are additional requirements, which induce T2D through the development of pancreatic β-cell failure [25,74,146].

### 2.3. Clinical Implications

#### 2.3.1. NASH and Fibrosis

Several studies have shown that T2D patients with NAFLD are at a high risk of NASH and cirrhosis [39,147,148,149]. Data from cross sectional studies [15,150,151,152,153] and longitudinal retrospective studies with sequential liver biopsies [154,155,156] clearly indicate that T2D strongly predicts fibrosis severity and progression in NAFLD patients. Consistently, two studies have demonstrated that poor glycemic control was associated with an increased risk of fibrosis in NASH [157,158].

Interestingly, one study showed that T2D and IR were strongly associated with NASH and severe fibrosis in patients with normal serum liver enzymes [159]. This finding provides further evidence to the clinical wisdom that “normal” serum liver enzyme levels are not a sufficient reason for excluding from liver biopsy those “high-risk” patients in whom advanced liver disease is strongly suggested by non-invasive evaluation. To this end, transient elastography and semi-quantitative ultrasound or non-invasive clinical scores (such as the US-FLI, the NAFLD fibrosis or the Fib4 scores) may be used in most patients with T2D [39,45,160,161].

#### 2.3.2. Cirrhosis and Hepatocellular Carcinoma

Many studies have reported T2D as an established risk factor for cirrhosis [162,163] and HCC [164,165,166]. Worryingly, a significant proportion of NAFLD patients with HCC have no evidence of cirrhosis [164], implying that they have escaped the normal surveillance strategies implemented in patients with cirrhosis of viral or alcoholic origin, and thus are diagnosed too late to receive radical treatment [167,168].

The presence of NAFLD among patients with T2D is also an important risk factor of increased all-cause and cause-specific mortality. Patients with T2D have an increased mortality risk from cirrhosis of any aetiology [39]. Accordingly, a recent cohort study showed that, compared to the age- and sex-matched general population, patients with T2D had a two- to three-fold higher risk of dying of non-viral and non-alcoholic chronic liver disease, largely attributable to NAFLD [169]. Consistently, a recent Scottish national retrospective cohort study reported that T2D was associated with an increased risk of hospital admissions or deaths for all common chronic liver diseases and, among them, NAFLD had the strongest association with T2D [170]. In agreement, a retrospective USA cohort study on 132 NAFLD patients found that T2D patients with NAFLD were at risk for the development of poor clinical outcomes, such as increased all-cause and liver-related mortality or morbidity after adjusting for potential confounding factors [162]. Finally, NAFLD was associated with a two-fold increased risk of all-cause mortality (mainly due to malignancy (33%), liver-related complications (19%) or ischemic heart disease (19%)) in a cohort study of 337 T2D patients followed-up for a mean period of 11 years [171].

#### 2.3.3. Atherosclerosis 

Accumulating evidence indicates that NAFLD is strongly associated not only with liver-related morbidity or mortality, but also with an excess risk of CVD, which is the most common cause of death in T2D [39]. Several studies have reported a strong association between NAFLD and early subclinical or advanced atherosclerosis among patients with and without T2D [172]. These findings have been further confirmed by multiple prospective studies that showed an increased risk of fatal and non-fatal CVD events in patients with and without T2D, independently of several cardiometabolic risk factors [39,172,173,174]. The association between NAFLD and risk of CVD mortality has been further supported by a milestone meta-analysis [17], although some recent follow-up studies are conflicting [172,175].

Emerging evidence also indicates that NAFLD is independently associated with the development of microvascular diabetic complications, *i.e.*, chronic kidney disease and advanced diabetic retinopathy [5].

Collectively, the above-mentioned studies convincingly show that T2D is strongly associated with an increased risk of progressive NAFLD and an excess risk of overall and cause-specific mortality, including not only liver-related but also CVD-related mortality. These findings fully support careful monitoring and screening for NAFLD and/or advanced fibrosis among patients with T2D.

## 3. HCV and Type 2 Diabetes

### 3.1 Epidemiology

#### 3.1.1. HCV and Diabetes: A Non-chance Association

The notion that cirrhosis is a potentially diabetogenic condition dates back to as early as 1906 [176]. More recently, such a view was confirmed in the pre-HBV and pre-HCV age [177]. It was more than 20 years ago that Allison *et al.*, [178] by comparing the rates of T2D among cirrhotic patients undergoing evaluation for liver transplantation, showed that T2D prevalence was 50% in patients with HCV-related *versus* 9% in those with non-HCV-related cirrhosis. Since that pioneering report, this topic has developed into a major line of research and, at the time of this writing, more than 1340 articles can be retrieved [179].

#### 3.1.2. The Burden

Licensing of oral direct acting antivirals (DAA), which deliver sustained virological response (SVR) rates >90%, has led to the revolutionary expectation that HCV infection will possibly be the first chronic viral infection totally eradicated [22]. However, such an inference is premature and, for the time being, HCV still infects from 150,000,000 to 185,000,000 people worldwide, namely up to 2.8% of the world population [180,181]. Moreover, in developing countries, the case-finding and management have not improved *in tandem*, suggesting that continued refinement of epidemiology, cost-utility models and targeted diagnostic strategies remain an unmet need [182]. Worldwide, chronic HCV infection remains a significant public health burden, given that it can lead to cirrhosis in approximately 15% to 20% of those infected within 20 years, resulting in end-stage liver disease and HCC [182]. In Europe, although the iatrogenic HCV transmission was enormously reduced over the last 20 years, transmission related to intravenous recreational drug use is on the increase, especially in Eastern Europe, and the high HCV prevalence in the migrant populations is a challenge [183]. Moreover, HCV-related morbidity and mortality are projected to increase in Europe until 2030 [183]. In the USA, up to 35% of patients on the liver-transplant waiting list are infected with HCV, and global HCV-associated mortality estimates approximate 500,000 deaths per year [184,185].

#### 3.1.3. Extra-Hepatic Manifestations of HCV Infection: Type 2 Diabetes

The clinical spectrum of chronic HCV infection is not limited to liver disease but also includes major extra-hepatic conditions, affecting eyes, salivary glands, skin, kidneys, genital tract, endocrine, neurologic, cardiovascular and immune systems (Figure 2) [8,37,38].

Among the extra-hepatic manifestations of HCV, a mutual and bi-directional relationship connects T2D with HCV infection. HCV infection is more common in patients with T2D than in those without T2D and, conversely, T2D abounds among patients with chronic HCV infection [177]. That said, however, the usual clinical scenario depicts a vignette in which, in predisposed individuals, HCV infection precedes and accelerates the development of new-onset T2D by approximately 10 years [38,186]. This finding suggests that HCV infection observed in T2D patients does not result from the risk of HCV infection associated with medical procedures in the highly medicalized T2D population but is the primary event which may adversely affect the subsequent development of T2D [187].

#### 3.1.4. Heterogeneity in the Distribution of HCV and Type 2 Diabetes and Differential Features of Hepatitis C-Associated Dysmetabolic Syndrome and MetS

There are 170,000,000 individuals with T2D worldwide, namely the same number of individuals with HCV infection [177]. However, HCV infection has undergone epidemiological diffusion in certain age groups and geographical areas as a result of specific lifestyle risk behaviors or transmission via medical practices, whereas T2D reaches its zenith among 45-to-64 year old individuals, particularly in obese and sedentary individuals [177]. Stated otherwise, the epidemiological distribution of HCV infection and T2D does not identify the same geographical areas and groups of individuals. Accordingly, screening campaigns to identify either HCV infection among T2D patients or T2D among those with HCV infection are not justifiable at this time and more accurate strategies are needed in screening selected cohorts of individuals [188].

Finally, it should be pointed out that while T2D is a prominent feature of the MetS which is bi-directionally associated with NAFLD [3], HCV infection is also associated with a specific hepatitis C-associated dysmetabolic syndrome (HCADS), which was first described by Lonardo *et al.* [189]. Table 1 schematically compares the main features of the MetS with those of the HCADS [3,7,168,190,191,192,193].

### 3.2. Pathophysiology

#### 3.2.1. HCV Increases T2D Risk via Insulin Resistance

Consistent with the development of new-onset T2D observed in the setting of NAFLD, HCV promotes a state of IR that leads, over time, to pancreatic beta-cell dysfunction, eventually culminating in the irreversible damage of such cells and the development of overt T2D [177].

#### 3.2.2. IR Associated with HCV: Antigens, Sites and Determinants

HCV antigens, such as the core protein, play a key role in determining post-receptor defects causing IR by interfering with the AKT signaling pathway via cytokines (such as TNF-α and interleukin-6) and the suppressors of cytokine signaling [194,195,196,197]. Strong evidence suggests that the site of IR is not only hepatic but also extra-hepatic [198], predominantly in the skeletal muscle, correlates with subcutaneous, rather than visceral adiposity, and is independent of liver fat content [199]. These findings conflict with the notion that HCV predominantly infects hepatocytes and suggest that either HCV-infected hepatocytes release a soluble mediator capable of inducing IR in skeletal muscles [38] or, alternatively, that HCV directly infects myocytes. This latter hypothesis appears to be conceptually sustainable based on the findings of a recent case-control study, which provided evidence for a significant association between inclusion body myositis and HCV infection [200].

#### 3.2.3. T2D in the Setting of the HCADS

T2D is not the only metabolic disease observed in the setting of HCV infection. Over time, several features of what is now alluded to as the HCADS have been increasingly identified. For example, hepatic steatosis, which is one of such features, was first identified as a distinct disease entity [7,21,201]. Data comparing hepatic steatosis due to varying viral (HIV-related) and non-viral (NAFLD) steatogenic disorders suggest that IR is a prominent feature specifically associated with HCV infection [202].

Over time, several features have been added to the initial description of the HCADS [203,204,205], which, presently, is deemed to characterize hyperuricemia, reversible hypocholesterolemia, IR, hypertension and visceral obesity [189]. Collectively, these dysmetabolic disorders may best be interpreted as a Darwinian survival strategy favoring the survival of HCV at the expenses of the host’s metabolism [189]. The finding of expanded visceral adipose tissue in patients with HCV infection is consistent with the hepatic and extra-hepatic origin of IR discussed above and prompts further research as to the potential ability of HCV infection to localize directly within adipocytes [206,207].

### 3.3. Clinical Implications

#### 3.3.1. Risk of Fibrosis

A consistent body of evidence supports the notion that T2D is closely associated with fibrosis in the setting of chronic HCV infection [188]. More recently, a large study conducted in USA in approximately 10,000 patients with hepatitis C found that age, sex, race, HCV genotype, HIV co-infection, alcohol abuse, antiviral therapy and T2D were independently associated with the risk of cirrhosis [208]. Moreover, a recent meta-analysis of 14 studies, involving 3659 participants with HCV infection, reported a significant association between IR and advanced hepatic fibrosis among patients with HCV genotype 1 infection but not among those with HCV genotype 3 [209]. These findings are consistent with those of previous studies reporting that IR was strongly associated with HCV genotypes 1 and 4 [210,211].

#### 3.3.2. Risk of Hepatocellular Carcinoma

Population-based studies fully support T2D being as an emerging risk factor for HCC [192]. In a recent meta-analysis, Dyal *et al.*, [193] have reported that concurrent T2D is strongly associated with an increased risk of HCC among chronic HCV patients. It may be argued, however, that, in these patients, T2D may either be a proxy of more advanced metabolic derangement which leads to excess fibrosis via NASH or that T2D *per se* exposes these individuals to higher risk of developing HCC via increased oxidative stress and hormonal changes (*e.g*., IR, increased IGF-1 and activation of the renin-angiotensin-aldosterone system) [193,212,213].

An Italian study conducted in 163 consecutive HCV-positive patients with cirrhosis followed-up for a median period of 10.7 years found that HCV genotype 1b was strongly associated with a higher risk of developing HCC [214].

Further studies are needed to control accurately for all viral and host’s confounders, such as genotype, obesity and ethnicity, given that an improved understanding of HCC risk factors may provide specific areas of targeted interventions to reduce HCC risk in chronic HCV patients [193].

#### 3.3.3. Risk of Atherosclerosis

The strong association between HCV infection and T2D development is one of the most important mechanisms that may lead to accelerated atherogenesis in chronic HCV patients [215]. Three studies showed that HCV infection is a strong risk factor for carotid subclinical atherosclerosis [216,217,218]. Consistent with the notion that HCV infection is a systemic disease, the risk of major CVD events is higher in patients with HCV infection than in HCV-negative controls, independently of traditional CVD risk factors and other potential confounding variables [219,220]. In a recent meta-analysis conducted on 22 studies, Petta *et al.* [191] showed that patients with chronic HCV infection had an increased risk of CVD-related morbidity and mortality, especially those with T2D and hypertension. On these grounds, all chronic HCV patients should be non-invasively screened for atherosclerosis [215].

## 4. Conclusions

**Figure 3:**
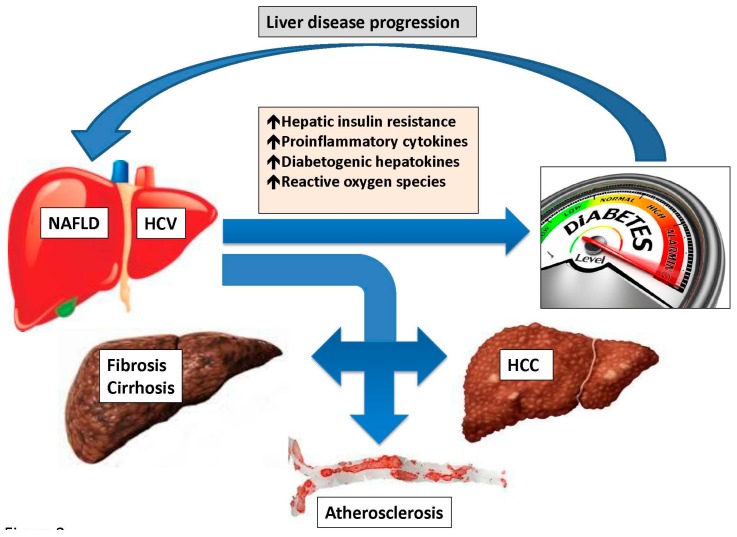
Non-alcoholic fatty liver disease, hepatitis C virus infection and type 2 diabetes: the “*vicious circle*”. The liver plays a pathogenic role in the development of type 2 diabetes both in the context of non-alcoholic fatty liver disease and hepatitis C virus infection through the development of systemic and hepatic insulin resistance, partly mediated by the release of multiple pro-inflammatory cytokines, diabetogenic hepatokines and reactive oxygen species. If left uncorrected, insulin resistance will eventually lead to progressive pancreatic beta cell failure in predisposed individuals. Moreover, the strong interconnection between type 2 diabetes and liver disease may result into a “vicious circle” [25] eventually leading to liver disease progression with an excess risk of liver-related, *i.e.*, cirrhosis and hepatocellular carcinoma (HCC), and cardiovascular complications, *i.e.*, atherosclerosis.

Among the “*four musketeers*” fighting for controlling glucose homeostasis, the liver is now in the spotlight of basic, epidemiological and clinical investigations (Figure 1). Indeed, by reviewing the role of HCV and NAFLD in the development of T2D, we found that there is a substantial body of evidence indicating that the liver plays a pathogenic role in T2D development and that the close inter-connections connecting T2D with liver disease may result in a “*vicious circle*” eventually leading to an excess risk of liver-related and CVD complications (Figure 3).

NAFLD and HCV infection are two multisystem diseases whose spectrum of clinical manifestations, seemingly as a result of their sharing hepatic steatosis and IR as prominent features (Figure 2) [205], tends to overlap more and more. Basic research is very active in the arena of NAFLD pathophysiology and extrapolation of notions from the NAFLD to the HCV research field appears to be justified and potentially fruitful [21].

However, several questions remain largely unanswered. For instance: is NAFLD treatment able to reduce the development of T2D and its major complications? Based on preliminary evidence [47,48] one may be tempted to answer affirmatively, though this remains to be fully proven by studies *ad hoc*. Does T2D impair SVR in the era of new direct-acting antivirals? While T2D was associated with a lower SVR rate following interferon-based therapy [7], regimens based on new direct-acting antiviral agents do not appear to be affected by coexisting T2D [221]. Moreover, whether HCV eradication may also have an impact on the future morbidity and mortality due to T2D is a clinically relevant and biologically plausible outcome. However, further studies with new direct-acting antivirals are needed to ultimately settle this issue [27].

In the meantime, it is important to underline that lifestyle changes are the mainstay of treatment for all patients with NAFLD and T2D [173,222]. It has been reported that a combination of educational, behavioral and motivational strategies may help patients with NAFLD in achieving lifestyle changes [223,224,225]. Preliminary evidence also suggests that body weight reduction may improve liver histology in those patients in whom HCV infection is associated with hepatic steatosis [226]. However, future studies are required to better define effective weight loss strategies in these patients.

## Figures and Tables

**Table 1:** Metabolic Syndrome *versus* Hepatitis C-Associated Dysmetabolic Syndrome (HCADS)—A comparison at a glance.

Criteria	Metabolic Syndrome	HCADS	Reference(s)
T2D	Yes	Yes	[3]
Hypertension	Yes	Yes	[3]
Visceral Obesity	Yes	Preliminary evidence suggests that HCV patients have abdominal fat distribution	[3]
Atherogenic dyslipidemia	Yes	Acquired, reversible hypocholesterolemia	[6]
Hepatic steatosis	Not included among diagnostic criteria but often found as a concurrent or precursor finding	In chronic HCV patients, steatosis is two- to three-fold more prevalent than in chronic hepatitides of other etiologies. HCV genotype 3 is associated with a higher prevalence and more severe steatosis	[3,6]
Hyperuricemia	Not included in diagnostic criteria but often associated on pathophysiological grounds	Strongly associated with severity of steatosis	[3,190]
Accelerated atherogenesis	Whether the full-blown MetS adds to the risk of its individual components, particularly T2D, is controversial	Individuals with HCV infection (particularly those with T2D and hypertension) have an excess of cardiovascular morbidity and mortality	[3,191]
HCC risk	Both the MetS and T2D increase the risk of HCC. This likely results via NAFLD/NASH even in non-cirrhotic livers	Concurrent T2D and chronic HCV infection lead to increased risk of HCC. Steatosis and overweight/obesity possibly play a role	[168,192,193]

## Abbreviations

The following abbreviations are used in this manuscript:

CCR-2: C-C chemokine receptor-2
CHD: coronary heart disease
chREBP: carbohydrate-responsive element-binding protein
CVD: cardiovascular disease
DAA: direct acting antivirals
DAG: diacylglycerol
DGAT2: diacylglycerolacyltransferase 2
DNL: de-novo lipogenesis
FA: fatty acids
FGF-21: fibroblast growth factor 21
FXR: farnesoid X receptor
HCC: hepatocellular carcinoma
HIF1α: hypoxia inducible factor 1α
HCV: hepatitis C virus
IR: insulin resistance
MCP-1: monocyte chemoattractant protein-1
MetS: metabolic syndrome
PNPLA3: patatin-like phospholipase domain-containing protein 3
PPAR-γ: peroxisome proliferator–activated receptor γ
ROS: reactive oxygen species
SREBP1c: sterol regulatory element binding protein 1c
T2D: type 2 diabetes
TLR-4: toll-like receptor 4
TNFα: tumor necrosis factor α
UCP-1: uncoupling protein-1
WAT: white adipose tissue
